# Roles of Extracellular Vesicles on the Progression and Metastasis of Hepatocellular Carcinoma

**DOI:** 10.3390/cells12141879

**Published:** 2023-07-18

**Authors:** Turner W. Seay, Zucai Suo

**Affiliations:** Department of Biomedical Sciences, College of Medicine, Florida State University, Tallahassee, FL 32306, USA

**Keywords:** enzymes, exosomes, extracellular vesicles, hepatocellular carcinoma, liver cancer, metastasis, circular RNAs

## Abstract

Liver cancer is a global health challenge as it is the third leading cause of cancer death worldwide. Hepatocellular carcinoma (HCC) is the most common type of liver cancer and is often found in liver cells, where it is associated with high morbidity and mortality rates. Recent studies have shown that extracellular vesicles (EVs) secreted by HCC cells play a critical role in HCC progression and metastasis. EVs contain proteins, nucleic acids, lipids, and metabolites as cargos. EVs derived from HCC cells can transfer oncogenic factors to surrounding cells leading to increased tumor growth, cell invasion, and angiogenesis. In this review, we summarize the roles that EVs play and the specific effects of their cargos on HCC progression and metastasis and identify potential therapeutic targets for HCC treatment.

## 1. Introduction

Liver cancer is a global disease, with an estimated incidence of >1 million cases per year by 2025. Liver cancer is a group of cancers with different incidences. Hepatocellular carcinoma (HCC) is the most common type of liver cancer, which starts in the liver cells. Less common types of liver cancer are called hepatobiliary cancers, which affect bile duct cells (cholangiocarcinoma), liver blood vessels (angiosarcoma), or gallbladder cells (gallbladder cancer). Hepatocellular carcinoma (HCC) accounts for ~90% of cases of liver cancer [1]. Many risk factors contribute to the development of HCC including infections with the hepatitis B virus (HBV), which accounts for about 50% of cases [2], and hepatitis C virus (HCV), which contributes as a risk factor with lesser HCC incidence [3] due to the success of antiviral drugs [4]. Other liver diseases such as cirrhosis, diabetes, or obesity-related non-alcoholic steatohepatitis (NASH) are growing risk factors for HCC [5].

**Figure 1:**
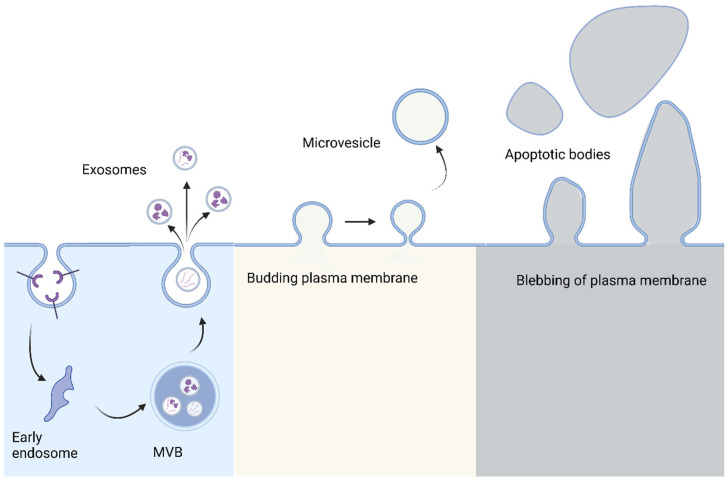
Biogenesis of different types of EVs. Exosomes are produced when the cell membrane buds inwards and creates an endosome. Exosomes are formed from internal budding of the endosome and multivesicular bodies (MVBs). Once the MVB docks to the membrane, the exosomes are released through exocytosis. Microvesicles and apoptotic bodies are formed from budding and blebbing of the plasma membrane, respectively, before being released from the membrane. Apoptotic bodies are the remnants of cells having undergone apoptosis, or programmed cell death.

Extracellular vesicles (EVs) are membrane-originated vesicles shed from all types of living cells and carry cargos of proteins, nucleic acids, lipids, metabolites, and even organelles from the parent cells [6,7]. The extracellular and vesicular properties of EVs had been recognized by researchers in the 1970s and the term EV was first used in 1970 [8]. EVs are grouped into exosomes, microvesicles, and apoptotic bodies as guided by the International Society of Extracellular Vesicles; they cannot replicate themselves and are generated by different mechanisms of biogenesis (Figure 1) [9]. In 1983, the laboratories of Rose Johnstone and Phil Stahl independently described exosomes as intraluminal vehicles to clear transferrin receptors from red blood cells upon maturation [10,11]. In general, exosomes with a diameter of 30–200 nm are produced through the invagination of the cell membrane and then the assembly of these vesicles into a multivesicular body, which are either cleared by the lysosomal pathway or fused to the cell membrane, releasing these exosomes into the extracellular milieu [12]. Microvesicles with a diameter of 0.2–1.0 μm originate from direct outward blebbing of the cell membrane. Apoptotic bodies range from 50 nm to 5 μm in diameter and are formed through a process termed apoptotic cell disassembly, which is characterized by a series of tightly regulated morphological steps including plasma membrane blebbing, apoptotic membrane protrusion formation, and fragmentation into apoptotic bodies. 

The importance of EVs in cancer biology stems from their molecular cargo of proteins, mRNAs, miRNAs, and DNAs. Another fascinating field capturing scientists’ imagination is the role of EVs as vehicles for the intercellular exchange of various materials. The intercellular exchange of membrane receptors [13] and miRNAs [14] are the starting points of the reinvention of the role of EVs as an intercellular communication mode in cancer. EVs have also been found to transport active nuclear receptors [15] and tumor suppressors [16], to name a few. Additionally, cargos in EVs are of huge interest as a source of biomarkers in various cancers [16,17], including HCC [18,19,20]. Herein, we will review the recent advances in the field of EVs as they pertain to HCC. We will also review the various roles of a few EV-encapsulated proteins and circular RNAs (circRNAs) and their effects on other proteins with enzymatic activities and pathways in HCC. If “exosomes” were used in a cited publication without proving experimental evidence for their MVB origin by the authors, we use “extracellular vesicles”, rather than “exosomes”, to describe their findings in this review. 

## 2. Effect of Glycolytic Enzymes as EV Cargos on HCC Progression

EV cargos contain a broad spectrum of proteins such as receptors, growth factors, enzymes, and others [6,7]. One of the important proteins found to be frequently downregulated in HCC cells is protein Rab20 [21]. This protein is a 26 kDa GTPase and has been shown to play a role in EV biogenesis. The downregulation of Rab20 in HCC cells causes them to release EVs that enhance these cells’ tumorgenicity in vitro and in vivo [21]. Moreover, the under-expression of Rab20 is found to be associated with under-expression of the triosephosphate isomerase 1 (TPI1) in EVs released by HCC cells with Rab20 knockdown [21]. TPI1 is a glycolytic enzyme which is positively correlated with Rab20 expression in EV-releasing cells. The restoration of TPI levels in EVs derived from cells with Rab20 knockdown suppresses the oncogenic effects of these EVs. TPI1 is involved in aerobic glycolysis, which is beneficial to the survival, growth, and proliferation of tumor cells [21]. The enhanced cell growth and motility are driven by the enhanced aerobic glycolysis induced by EVs with reduced TPI1 [21]. In addition, EVs from highly metastatic HCC cells, MHCC97H, stimulate migration and invasion of low metastatic HCC cells [22]. Knock-down of Rab27a, a 25 kDa GTPase, in MHCC97H cells inhibits MHCC97H-derived EV secretion and promotes migration, chemotaxis, and invasion of these cells [22]. Blocking EV secretion in these cells increases lung and intrahepatic metastasis in vivo, while injection of MHCC97H cell-derived EVs promotes the recurrence of HLE tumors in the liver of experimental animals [22].

The glycolysis process is augmented in cancer cells to support the anabolic requirements to maintain homeostasis in response to various stresses. Another protein that is important in the process is pyruvate kinase M2 isoform (PKM2), which catalyzes the final step of glycolysis. PKM2 is highly expressed in cancer cells, and it promotes tumor anabolic metabolism, survival, and proliferation [23]. PKM2 was found to be incorporated into the cargos of EVs and it contributes to the development of HCC via intercellular communication between HCC and monocyte/macrophage [23]. HCC induces the differentiation of monocytes into infiltrating macrophages via EV-PKM2 promoting HCC development [23].

Enolase is a metalloenzyme that catalyzes the conversion of 2-phosphoglycerate to phosphoenolpyruvate in the glycolysis process. Its enzymatic reaction is reversible and depends on the availability of 2-phosphoglycerate and phosphoenolpyruvate in the environment. There are three isotypes of this enzyme in mammals: α-enolase (ENO1) expressed in most tissues, β-enolase (ENO-3) expressed in muscle tissue, and γ-enolase (ENO-2) expressed in neural tissues. The three isotypes form homodimers with two magnesium ions bound at their active sites [24]. ENO1 is important because, in addition to glucose metabolism, it plays a role in autoimmune response, hypoxia endurance, and growth regulation. ENO1 also plays a role in tumorigenesis and cancer progression in various cancer types, including HCC [25]. It is considered a biomarker for HCC, and is highly expressed in HCC cells with high metastatic potential and metastatic tumor tissues [25]. Highly metastatic HCC cells shed ENO1 within the cargos of EVs (EV-ENO1). EV-ENO1 can be transported to nonmetastatic HCC cells, conferring aggressive and metastatic properties to low ENO1-expressing HCC cells [26]. ENO1 confers such characteristics by upregulating the expression of integrin α6β4 and activating the FAK/Src-p38MAPK pathway. As such, EV-ENO1 promotes the growth and metastasis of HCC [26].

## 3. Caspase-3 Activators Incorporated into EVs Affect HCC Development

**Figure 2:**
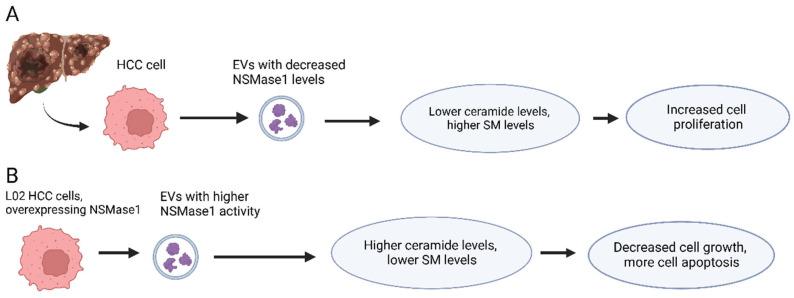
Effects of NSMase1 level in extracellular vesicles released from HCC Cells. (**A**) HCC cells in patients frequently produce extracellular vesicles with decreased NSMase1 level, leading to lower ceramide levels and higher sphingomyelin levels which result in increased HCC cell proliferation. (**B**) When HCC cells overexpress NSMase1 their extracellular vesicles have higher NSMase activity leading to higher ceramide levels and lower sphingomyelin leading to a decrease in HCC cell growth and increased apoptosis.

Caspase-3, a crucial player in programmed cell death (apoptosis), is a frequently activated death protease catalyzing the specific cleavage of many cellular proteins [27]. Caspase-3 deficiency in tumor cells or tumor stroma causes significant tumor sensitivity to radiotherapy in experimental animals [28]. Activated caspase-3 levels in tumor tissues of HCC patients are associated with a higher rate of recurrence and death [28]. Caspase-3 is activated by neutral sphingomyelinase 1 (NSMase1). Sphingomyelinase (SMase) cleaves the phosphodiester bond in sphingomyelin (SM) to form ceramide (Cer) and phosphocholine. Ceramide acts as a second messenger in activating the apoptotic cascade [29]. Ceramide levels have also been shown to be greatly reduced in HCC tissues [30]. HCC tissues have significantly low expression of NSMase1, which contributes to poor survival of HCC patients (Figure 2A) [31]. This NSMase1 down-regulation in HCC tissue leads to the accumulation of SM and reduced Cer levels, leading to an increased SM/Cer ratio [31]. Both NSMase1 expression level and NSMase activity have been found to decrease in EVs derived from HCC tissues [31]. Overexpression of NSMase1 in HCC cells L02 results in high levels of NSMase activity in EVs isolated from the culture medium of these cells [31]. NSMase1 enriched EVs inhibit cell growth and induce apoptosis of HCC cells by decreasing the ratio of SM/Cer (Figure 2B) [31]. It is believed that NSMase1, through its ability to cleave SM into Cer, acts as a key factor in the activation of the Caspase-3 apoptotic pathway in HCC cells by increasing Cer levels. 

## 4. EV-Bound p120 Catenin Effects on HCC Proliferation and Metastasis

The function of many organs is dependent on the generation of epithelium. The transition from unconnected, unpolarized cells to connected, polarized epithelial cells requires the assembly of cell junctions. There are three types of cell junctions that support epithelium integrity, and they are adherens junctions, tight junctions, and desmosomes. p120 catenin (p120ctn) is a member of the Armadillo family and a component of the cadherin–catenin complex in the adherens junction. p120ctn performs many functions depending upon its subcellular localization. Besides cell–cell adhesion, p120ctn stabilizes cadherins at the cell membrane by modulating cadherin membrane trafficking and degradation, regulates the activation of small Rho GTPases in the cytoplasm, and modulates nuclear transcription. 

**Figure 3:**
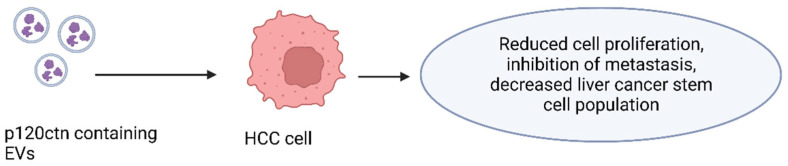
Effects of p120ctn containing EVs on HCC cells. When extracellular vesicles containing higher levels of p120ctn were introduced to HCC cells they had reduced cell proliferation, inhibition of metastasis, and a lower cancer stem cell population.

The expression pattern of p120ctn is abnormal in many human tumors [32,33]. p120ctn is absent or altered in most human tumors, and its derangement is often correlated with a poor prognosis. p120ctn can act as a proto-oncogene or as an invasion suppressor. Loss of p120ctn results in decreased E-cadherin levels. E-cadherin is frequently down-regulated in epithelial cancers, in which it acts as a tumor suppressor [34,35,36]. Cheng et al. reported that p120ctn incorporated into EVs, secreted by liver cancer cells, inhibits the proliferation and metastasis of hepatoma cells and the expansion of liver cancer stem cells (Figure 3) [37]. The EV-transmitted p120ctn has also been found to thwart HCC progression by inhibiting the STAT3 pathway, suggesting that p120ctn-containing EVs derived from cancer cells may be used as a therapeutic agent to treat liver cancer [37].

Another enzyme that plays an important role in β-Catenin signaling is the Vacuolar Protein Sorting 4 Homolog A (Vps4A). Vps4A is a member of the AAA protein family, which are ATPases associated with diverse cellular activities. Based on functional studies, Vps4A is associated with endosomal compartments in humans and plays a role in intracellular protein trafficking, such as the Vps4 protein in yeast. Han et al. [38] investigated the role of Vps4A in sorting proteins into extracellular vesicles of HCC cells. They performed mass spectrometry analysis for the protein complex associated with Vps4A upon immunoprecipitation, confirming that Vps4A is associated with β-catenin and Charged Multivesicular Body Protein 4B (CHMP4B). The authors concluded that through this interaction, Vpas4A promotes the plasma membrane localization and release of β-catenin via EVs [38]. The release of β-catenin into EVs suppresses β-catenin signaling within the cells. Furthermore, the authors investigated the effect of silencing Vps4A or CHMP4B on the plasma membrane localization and release of β-catenin through EVs [38]. Silencing Vps4A or CHMP4B decreases both plasma membrane localization and EV sorting of β-catenin, leading to enhanced signaling of β-catenin in the cells, where overexpression of these proteins resulted in decreased β-catenin signaling due to the increased secretion of β-catenin via EVs. The authors also investigated the relation between Vps4A expression in HCC tissues and the level of β-catenin loaded into EVs in patients with metastatic HCC [38]. The authors found that EV β-catenin expression in metastatic HCC patients is significantly lower than in patients with nonmetastatic tumors. Prior to this work, the authors showed that Vpas4A, as a key regulator of EV biogenesis, is frequently downregulated in HCC tissues and the downregulation is associated with tumor progression and metastasis [39]. Their in vitro experiments showed that Vps4A inhibits the growth, colony formation, migration, and invasion of HCC cells. The authors also studied the involvement of Vps4A in suppressing the bioactivity of EVs and characterized its ability to weaken the cell response to EVs [39]. Vps4A is found to facilitate the incorporation of oncogenic miRNAs into EVs, and the authors concluded that Vps4A is a tumor suppressor as mentioned above [39].

## 5. Upregulation of the 14-3-3ζ Protein Impairs Antitumor Functions

The antitumor functions of tumor-infiltrating T lymphocytes (TILs) are often inhibited in HCC, but the inhibitory mechanisms are not fully understood. Wang et al. found that the 14-3-3ζ protein, a negative regulator of the cell cycle, is incorporated into EVs derived from HCC cells [40]. Identified as a p53-inducible gene product, the 14-3-3ζ protein is responsible for controlling cell cycle checkpoints after DNA damage. Decreased expression of 14-3-3ζ in several cancers suggests a negative regulatory role of 14-3-3ζ in the cell cycle, which is compromised during tumorigenesis [41]. The 14-3-3ζ protein, however, has been shown to be upregulated in HCC cells and enhances invasion when under hypoxic condition [42]. Interestingly, 14-3-3ζ is also overexpressed in TILs in HCC [40]. The upregulation of 14-3-3ζ results in impaired activation, proliferation, and antitumor functions of TILs in HCC [40]. TIL cells with high levels of 14-3-3ζ have a high frequency of exhausted phenotypes as measured by the inhibitory receptors PD-1, TIM-3, LAG3, and CTL-4 [40]. Thus, Wang et al. concluded that the antitumor functions of TILs are inhibited once the TILs uptake the EVs carrying the 14-3-3ζ protein and 14-3-3ζ can be transmitted from HCC cells to T cells through EVs [40]. 

## 6. Impact of Lysyl Oxidase-Containing EVs on HCC Proliferation and Migration

**Figure 4:**
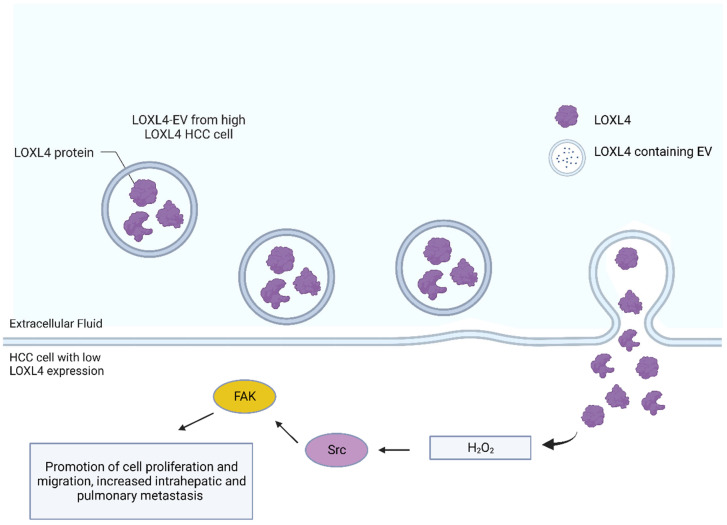
LOXL4-containing extracellular vesicles from high LOXL4-expressing HCC cells travel to low LOXL4-expressing HCC cells. LOXL4 then activates the FAK/Src pathway through hydrogen peroxide leading to promotion of cell proliferation and migration as well as increased intrahepatic and pulmonary metastasis.

Lysyl oxidase (LOX), a copper-containing amine oxidase, is a member of a heterogeneous family of enzymes that oxidize primary amine substrates to reactive aldehydes. The enzyme is known for its function of extracellular catalysis to form lysine-derived cross links in fibrillar collagens and elastins. Lysyl oxidases have been found to contribute to a spectrum of biological functions such as developmental regulation, tumor suppression, cell motility, and cellular senescence. LOX can be found in intracellular and intranuclear locations. Other LOX functions such as a potential role in cell adhesion and cell growth control can be determined by the conserved domains of LOX, such as the cytokine receptor-like domain that is shared by all LOXs and by multiple scavenger receptor cysteine-rich (SRCR) domains present in LOX-like 2 (LOXL2), 3 (LOXL3), and 4 (LOXL4) [43]. Furthermore, these functions may be carried out in a temporally and spatially regulated fashion [43]. LOXL4 is known to be upregulated in HCC tumor tissues and such upregulation is associated with a poor prognosis for HCC patients. LOXL4 has even been shown to play an important role in hepatocarcinogenesis by creating a microenvironment that is immunosuppressed during tumor development [44]. In vitro, LOXL4 knockdown inhibits HCC cell migration and invasion, and its upregulation in vivo promotes intrahepatic and pulmonary metastasis of HCC [45]. Interestingly, LOXL4 is present in the cargo of HCC-secreted EVs, and HCC cells that have lower expression levels of LOXL4 are targeted by the EVs secreted by HCC cells with higher LOXL4 expression (LOXL4-EVs) [45]. These LOXL4-EVs transfer LOXL4 to the HCC cells with lower expression levels of LOXL4 and promote migration of the cells by activating the FAK/Src pathway via its amine oxidase activity through the hydrogen peroxide-mediated mechanism (Figure 4) [45]. These LOXL4-EVs also transfer LOXL4 to human umbilical vein endothelial cells to promote angiogenesis via the paracrine mechanism [45]. 

## 7. Targeting the Complement System in HCC Progression

The complement system contains a large number of plasma proteins that react with one another to opsonize pathogens and induce a series of inflammatory responses that help fight various infections. Many complement proteins are proteases that are themselves activated by proteolytic cleavage [46]. The system is an essential component of the innate immune system that eliminates pathogens and altered host cells such as cancer cells. The complement system links innate and adaptive immune responses. There are two types of complement regulators that protect the tissue from complement-mediated injuries. The regulators are soluble and membrane-bound complement regulators. Complement factor H (CFH) is one of the complement regulators that inhibits the alternative pathway of the complement activation in blood and cell surfaces. Cancer cells utilize CFH to escape immune surveillance and use it as an invasion factor. Mao et al. showed that metastatic HCC cells shed EVs enriched with CFH [47]. These EVs stimulate HCC cell growth, migration, and invasiveness and enhance liver tumor formation and metastasis in mice. Treating EVs containing CFH with anti-CFH antibodies significantly inhibits the abovementioned stimulation effects of the EVs [47]. These facts indicate that the EVs enriched with CFH protect HCC cells from complement system attack, leading to increased tumorgenicity and metastasis. The role of CFH in HCC is controversial, however, as other studies have shown that the absence of CFH can lead to chronic alternative pathway activation and thus development of HCC [48]. While the role of this protein in HCC is up for debate, recent studies show the importance of further investigation of its impact on HCC.

## 8. Encapsulated Circular RNAs Affect the Progression of HCC

Circular RNAs are a group of single-stranded RNA molecules that are categorized by having a covalently closed continuous loop structure with no 5′ and 3′ ends and are caused by RNA splicing events [49,50,51]. These RNA molecules are mostly found in the cytoplasm and are shown to act as miRNA sponges by binding to miRNAs and inhibiting their function. CircRNAs have been shown to have many effects on HCC including invasion, proliferation, metastasis, and immune suppression [52]. CircRNAs do not have 5′ and 3′ ends and are resistant to exonuclease-mediated degradation, leading to higher stability than most linear RNA in cells [53]. Extracellular vesicles are excellent vehicles to carry and deliver circRNAs between cells for intercellular signaling. Exosomal circRNAs are utilized by HCC cells to enhance growth, metastasis, and immunosuppression. Z. Hu et al. have identified a novel circRNA, circCCAR1, and showed that through a circCCAR1/miR-127-5p/WTAP feedback loop, HCC growth and metastasis increases in vitro and in vivo [54]. This circRNA has also been shown to be released by HCC cells as one of the cargos of EVs, which leads to CD8+ T-cell dysfunction in HCC [54]. Other exosomal circRNAs have been shown to also promote HCC progression. Y. Zhou et al. showed that exosomal circZFR released from cancer-associated fibroblasts enhances HCC cell chemoresistance [55]. These studies open the floodgate to search and find other circRNAs contained by HCC-secreted EVs and establish new targets for combatting chemoresistance and HCC progression. For example, it is possible to load DNA or RNA oligomers into HCC-derived extracellular vesicles through cell-penetrating peptides [56] in order to bind to targeted circRNAs, resulting in their functional inhibition and possible destruction in vivo.

## 9. Conclusions

Extracellular vesicles contain various circRNAs and proteins including enzymes that could modulate the tumor microenvironment. Tumor cells secrete EVs to assist in creating a favorable microenvironment to help tumor cell survival, growth, and metastasis. EVs containing circRNAs and enzymes can pass these cargos between cancer cells and assist tumor progression by stimulating the growth, proliferation, and invasiveness of cancer cells. These delivered cargos also contribute to the activation of the process of angiogenesis, which is essential to tumor growth. Lastly, these EV cargos contribute to the inhibition of the host response to the existence of cancer cells, providing cancer cells the opportunity to escape immunosurveillance. As a result of these functions and others beyond the scope of this review, EVs are major players in the progression and metastasis of HCC. To help readers to read and follow this review, Table 1 summarizes the sources, targets, cell and animal models, clinical samples, and cited publications for the discussed proteins and circular RNAs encapsulated by HCC-derived EVs. The cited references for each discussed topic in this review are listed in Table 2.

## Figures and Tables

**Table 1:** Exosomal cargos relevant to HCC and their cellular targets, cell and animal models, and clinical samples.

Protein	Source	Target	Cell Model/Line	Animal Model	Clinical Samples	Reference
Rab20	HCC cells	Secretory pathway	MIHA cell line	BALB/C nude mice	Obtained from Queen Mary Hospital, Hong Kong	[21]
PKM2	HCC cells	Tumor microenvironment	Derived from C57BL/6 mice bone marrow, blood, and liver	C57BL/6 mice	Obtained from Zhongshan Hospital, Xiamen University	[23]
ENO1	HCC cells	Integrin α6β4 and the FAK/Src-p38MAPK pathway	HepG2, LO2, MHCC97L, MHCC97H, and HCCLM3	BALB/c-nu/nu nude mice	Not used	[26]
NSMase1	HCC cells	Sphingomyelin (SM)	SMMC7721 and LO2	Not used	Obtained from Affiliated Hospital of Guilin Medical University	[31]
p120ctn	Liver cancer cells	STAT3 pathway	CSQT-2, HCCLM3 (LM3), 7701, and 7702	Male nude mice	Obtained from Eastern Hepatobiliary Surgery Hospital	[37]
Vps4A	HCC cells	CHMP4B and β-catenin	SMMC-7721, MHCC97H, and Huh-7	Not used	Obtained from Sun Yatsen Memorial Hospital, Sun Yat-sen University	[38,39]
14-3-3ζ protein	HCC cells	Tumor-infiltrating T lymphocytes (TILs)	Human PBTC cells	Male C57BL/6 mice	Obtained from First Affiliated Hospital of Nanjing Medical University	[40]
LOXL4	HCC cells	FAK/Src pathway	SK-Hep1, SUN-423, Hep3B, Huh7, SMMC-7721, MHCC-97 L, and MHCC-LM3	Male BALB/c-nu/nu mice	Obtained from Ren Ji Hospital, School of Medicine, Shanghai Jiao Tong University	[45]
CFH	HCC cells	Complement and immune system	Hep3B, Huh7 PLC/PRF/5, human 293FT, murine Hepa1-6, HLE, MHCC97L, and MHCCLM3	Male BALB/cAnN-nu mice, BALB/c nude mice, and C57BL/6N mice	Not used	[47]
circRNAs	HCC cells	miRNAs	LO2, Huh7, and MHCC97L	Not used	Obtained from First People’s Hospital of Kunming City	[51,52]

**Table 2:** Cited publications in discussed topics.

Topics	Relevant References
Effect of glycolytic enzymes as EV cargos on HCC progression	[21,22,23,24,25,26]
Caspase-3 activators incorporated into EVs affect HCC development	[27,28,29,30,31]
EV-bound p120 catenin effects on HCC proliferation and metastasis	[32,33,34,35,36,37,38,39]
Upregulation of the 14-3-3ζ protein impairs antitumor functions	[40,41,42]
Impact of lysyl oxidase-containing EVs on HCC proliferation and migration	[43,44,45]
Targeting the complement system in HCC progression	[46,47,48]
Encapsulated circular RNAs affect the progression of HCC	[49,50,51,52,53,54,55,56]

## Data Availability

No new data were created in this study. Data sharing is not applicable to this article.
